# Characterization and targeting of phosphatidylinositol-3 kinase (PI3K) and mammalian target of rapamycin (mTOR) in renal cell cancer

**DOI:** 10.1186/1479-5876-9-133

**Published:** 2011-08-11

**Authors:** Aymen A Elfiky, Saadia A Aziz, Patricia J Conrad, Summar Siddiqui, Wolfgang Hackl, Michel Maira, Camp L Robert, Harriet M Kluger

**Affiliations:** 1 Division of Medical Oncology, Dana-Farber Cancer Institute, 450 Brookline Ave., Boston, MA 02215, United States of America; 2 Section of Medical Oncology, Yale Cancer Center, Yale University, 333 Cedar St, New Haven, CT 06520, United States of America; 3 Department of Pathology, Yale University, 333 Cedar St, New Haven, CT 06520, United States of America; 4 Novartis Institutes for Biomedical Research, AG CH-4002, Basel, Switzerland

## Abstract

**Background:**

PI3K and mTOR are key components of signal transduction pathways critical for cell survival. Numerous PI3K inhibitors have entered clinical trials, while mTOR is the target of approved drugs for metastatic renal cell carcinoma (RCC). We characterized expression of p85 and p110α PI3K subunits and mTOR in RCC specimens and assessed pharmacologic co-targeting of these molecules *in vitro*.

**Methods:**

We employed tissue microarrays containing 330 nephrectomy cases using a novel immunofluorescence-based method of Automated Quantitative Analysis (AQUA) of *in situ *protein expression. In RCC cell lines we assessed synergism between PI3K and mTOR inhibitors and activity of NVP-BEZ235, which co-targets PI3K and mTOR.

**Results:**

p85 expression was associated with high stage and grade (*P *< 0.0001 for both). High p85 and high mTOR expression were strongly associated with decreased survival, and high p85 was independently prognostic on multi-variable analysis. Strong co-expression of both PI3K subunits and mTOR was found in the human specimens. The PI3K inhibitor LY294002 and rapamycin were highly synergistic in all six RCC cell lines studied. Similar synergism was seen with all rapamycin concentrations used. NVP-BEZ235 inhibited RCC cell growth *in vitro *with IC_50_s in the low ηM range and resultant PARP cleavage.

**Conclusions:**

High PI3K and mTOR expression in RCC defines populations with decreased survival, suggesting that they are good drug targets in RCC. These targets tend to be co-expressed, and co-targeting these molecules is synergistic. NVP-BEZ235 is active in RCC cells *in vitro; *suggesting that concurrent PI3K and mTOR targeting in RCC warrants further investigation.

## Background

Renal cell carcinoma (RCC) is among the ten leading causes of cancer-related deaths, and the incidence has been increasing by approximately 2% per year [1-4]. RCC is typically resistant to chemotherapy and radiation therapy. The five-year survival rate is 90.8% for localized RCC (confined to primary site), 63.3% for cases with regional disease, and 11.1% in patients with distant metastases [5]. The immunogenicity of RCC has been the basis for use of cytokines such as interleukin-2 and interferon for metastatic RCC, which benefit about 15% of patients [6,7]. Alternative drugs are needed for patients who are not responsive and/or are intolerant to these therapies.

A growing understanding of the pathogenesis of RCC has enabled us to identify factors pertinent to development of RCC-targeting therapies. The discovery of VHL tumor-suppressor gene inactivation and consequent hypoxia-induced factor (HIF) activation of genes and downstream pathways important to tumor progression, have provided the impetus for development of new agents that target angiogenesis and proliferation pathways. Specifically, therapies that have demonstrated benefit in metastatic RCC include the small molecule tyrosine kinase inhibitors sunitinib, sorafenib and pazopanib [8-10], the anti-VEGF antibody bevacizumab [11], temsirolimus and everolimus, inhibitors of mTOR, which has been implicated in HIF transcription [12]. Although these new agents improve progression free survival, none have shown a statistically significant improvement in overall survival. In effect none are curative, and duration of response is often limited.

The PI3K pathway is activated and/or up-regulated in cancers, and plays a critical role in tumor progression [13,14]. There are three classes of PI3Ks; each has its own substrate specificity [15,16]. Class I_A _PI3Ks, the most widely implicated in cancer, primarily phosphorylate phosphatidylinositol-4,5-bisphosphate to generate the second messenger phosphatidylinositol-3,4,5-trisphosphate. This enzyme is a heterodimer consisting of a p85 regulatory and a p110 catalytic subunit. Class I_A _PI3K is activated by receptor tyrosine kinase (RTK) signaling [17,18]. Binding of p85 to activated RTKs serves both to recruit the p85-p110 heterodimer to the plasma membrane, where its substrate phosphatidylinositol-4,5-bisphosphate resides, and to relieve basal inhibition of p110α by p85. Downstream mediators, including Akt and PDK1, directly bind to phosphatidylinositol-3,4,5-trisphosphate. Akt phosphorylates several cellular proteins, including GSK3, GSK3ß, FOXO transcription factors, MDM2, and BAD, to facilitate cell survival and cell cycle entry [15]. Akt phosphorylation also results in activation of the mTOR/raptor complex, which regulates protein synthesis, cell growth, and proliferation [19]. There are two distinct functional mTOR complexes, mTORC1 and mTORC2. mTORC1 (rapamycin sensitive) consists of mTOR and Raptor, and its activation results in phosphorylation of p70S6 and 4E-BP1. mTORC2 consists of mTOR and the rapamycin-insensitive companion of mTOR (Rictor), and causes Akt phosphorylation. Akt promotes protein synthesis and cell growth by alleviating TSC1/2 suppression of mTOR, allowing the latter to act as part of the mTOR-raptor complex on 4EBP1 and S6 kinases.

Activation of the PI3K pathway in cancers has been demonstrated in numerous studies. The two most common mutations are of p110α (*PIK3CA*) and loss of the tumor suppressor *PTEN*. Amplification of *PIK3CA *and Akt are occasionally observed in epithelial cancers [15]. Recently, high expression of the PI3K/p110γ isoform was implicated in pancreatic adenocarcinoma progression [20]. There is specific evidence of PI3K pathway activation in RCC; it is constitutively activated in RCC cells regardless of VHL status, and activation is tumor specific [21]. Activation of mTOR can also up-regulate HIF gene expression, which, in patients with VHL mutations, can magnify HIF accumulation and expression of HIF-inducible genes.

In RCC, data from moderate sized studies support activation of the mTOR signaling pathway. Immunostained tissue microarray sections of 150 RCCs showed significantly higher expression of phosphorylated p70S6K (p-p70S6K), phosphorylated-mTOR (p-mTOR) and phosphorylated Akt (p-Akt) compared to normal kidney, p < 0.05 [22]. Additionally, Robb et. al found strong co-expression of phosphorylated-S6 (p-S6) and p-mTOR in 14 of 29 clear cell carcinomas [23]. Significantly decreased mean disease-free survival was observed when caveolin was co-expressed p-AKT, p-mTOR, p-S6 and phosphorylated-4EBP1 [24]. Therefore, inhibition of mTOR has the potential to inhibit tumor progression at multiple levels, and along with PI3K inhibition is particularly attractive for development for RCC treatment.

Despite the literature demonstrating the importance of PI3K and mTOR in RCC pathogenesis, there is limited information on total protein expression and co-expression in large cohort RCC tumor studies in the context of patient survival. A previous meta-analysis of mRNA expression microarrays revealed signature alternations in the PI3K/AKT pathway that are associated with tumor versus benign renal tissue [25]. Merseberger et. al determined expression patterns of PI3K, PTEN, p-Akt for possible prognostic value in 176 RCC cases, and found that activation of the PI3K pathway is associated with adverse clinical outcome [26]. In a more recent study, metastatic RCC samples from 132 patients and a subset of 25 matched primary RCC specimens were stained for PI3K, PTEN, p-Akt, p-mTOR, and p70S6. p-mTOR was associated with decreased survival [27].

The relevance of the PI3K/Akt/mTOR signaling pathway in RCC is the focus of ongoing research. Single-agent mTOR inhibitors have some efficacy in RCC, and co-targeting additional PI3K pathway members along with mTOR might be a valuable strategy for overcoming the escape mechanisms that can limit activity of mTOR inhibitors. Seeing that PI3K inhibitors are currently in clinical development, our purpose was to assess co-expression of PI3K subunits, p110α and p85, and mTOR in RCC tumors in a quantitative fashion and study pharmacological co-inhibition of these targets *in vitro*. To thoroughly assess co-expression of mTOR and PI3K subunits in a quantitative fashion, we employed a new method of automated, quantitative analysis (AQUA) of *in situ *protein expression, which has been validated and used in a number of previous studies [28,29]. Expression of mTOR and PI3K, p85 and p110α subunits was assessed in a large cohort of human specimens and we determined associations with standard clinical/pathological variables. We further studied co-targeting these molecules in RCC cell lines, and assessed the effects on cell growth and apoptosis using a clinical quality compound, NVP-BEZ235.

## Methods

### Tissue Microarray (TMA) Construction

Briefly, representative regions were selected for coring by pathologists based on the corresponding H&E-stained full sections. The tissue microarray was constructed with single 0.6 mm-diameter cores of each case spaced 0.8 mm apart in a grid format using a Tissue Microarrayer (Beecher Instruments, Sun Prairie, WI). The tissue microarray block was then cut into 5 μm sections with a microtome, adhered to the slide by an adhesive tape-transfer method (Instrumedics, Inc., Hackensack, NJ) and UV crosslinked. TMAs were constructed using RCC cores from 330 patients. Tumors were represented by two cores from different areas of the specimen. Specimens and clinical information were collected with approval of a Yale University Institutional Review Board. Histological subtypes included clear cell (71%), papillary (14%), chromophobe (2%), mixed histology (4%), oncocytomas (6%), and sarcomatoid tumors (3%). Oncocytomas were excluded from survival analyses given that they have low metastatic potential and are curable by nephrectomy. Eight percent had stage II and III disease, 56% had stage I and 28% had stage IV disease. 12% were Fuhrman nuclear grade I, 52% grade II, 27% grade III and 9% grade IV. Specimens were resected between 1987 and 1999; follow-up time was 2-240 months (median-89.7). Age at diagnosis was 25-87 years (median-63). No patients were treated with sunitinib, sorafenib, pazopanib, bevacizumab, everolimus or temsirolimus, although a few were previously treated with interferon or interleukin-2 in the metastatic setting. Performance status, LDH, hemoglobin and calcium levels were unavailable.

### Immunofluorescence

One set of two slides (each containing a core from different areas of tumor for each patient) was stained individually for the three target markers, p85 and p110α PI3K subunits, and mTOR. Antibody validation was conducted by immunoblots to verify presence of a single band of the appropriate size (not shown). AQUA staining was performed as described [30]. Slides were incubated with mouse monoclonal anti-human PI3K p85, (BD transduction Laboratories, Franklin Lakes, NJ) at 1:50, rabbit anti-human PI3K p110α, clone C73F8 (Cell Signaling Technology, Danvers, MA) at 1:200 or rabbit monoclonal anti-human mTOR, clone 7C10 at 1:40, (Cell Signaling Technology). Goat anti-mouse (or anti-rabbit) HRP-decorated polymer backbone (Envision, Dako North America, Carpinteria, CA) was used as a secondary reagent. To create a tumor mask, slides were simultaneously incubated with rabbit (for p85) or mouse (for p110α and mTOR) anti-cytokeratin (Dako) at 1:100, and visualized with an appropriate secondary antibody conjugated to Alexa 488 (Molecular Probes, Inc., Eugene, OR). The target antibody was visualized with Cy5-tyramide (Perkin-Elmer, Boston, MA, and mounted with ProLong Gold antifade reagent with 4, 6-diamidine-2-phenylindole (DAPI) (Invitrogen, Carlsbad, CA). To verify that there was no background staining from the Alexa 488, slides were stained with and without Cy5 tyramide.

### Automated Image Acquisition and Analysis (AQUA)

Images were acquired and analyzed using extensively described algorithms [30]. Briefly, monochromatic, high-resolution (1024 × 1024 pixel) images were obtained of each histospot. Tumor was distinguished from stroma by cytokeratin/streptavidin signal. Cell surface coalescence of cytokeratin was used to localize membranes and DAPI to identify nuclei. The target signal (p85, p110α or mTOR) from the pixels within the cytoplasm was normalized to area of tumor mask and scored on a scale of 0-255 (the AQUA score). Histospots were excluded if the tumor mask represented < 3% of the histospot area.

### Statistical Analysis

Statview and JMP 5.0 software were used (SAS Institute, Cary, NC). AQUA scores for replicate tumor cores were averaged. Prognostic significance of parameters was assessed using the Cox proportional hazards model with RCC-specific survival as an endpoint. Associations between continuous AQUA scores of the target and clinical and pathological parameters were assessed using ANOVA. For demonstrating survival analyses, continuous target AQUA scores were divided into quartiles and survival curves were generated using the Kaplan-Meier method, with significance evaluated using the Mantel-Cox log-rank test.

### Human Cell Lines

A498, ACHN, Caki-1, Caki-2, 769-P, and 786-0 cells were obtained from American Type Culture Collection and maintained per the supplier's instructions (Manassas, VA).

### Viability and Synergism Studies

At a density of 10^3^, cells were plated in triplicate in 96 well plates with growth medium and allowed to adhere overnight. The PI3K inhibitor, LY294002 (LC Laboratories, Woburn MA), was used alone and in combination with the mTORC1 inhibitor, Rapamycin (LC Laboratories), at 5-25 μmol/L and 0.02-0.5 μmol/L, respectively for 48 hours. NVP-BEZ235 was studied alone at concentrations of 10-500 ηmol/L for 48 hours. The relative number of viable cells was assessed by the luminometric Cell-Titer Glo assay (Promega), and luminescent quantification was measured using a Viktor plate reader (Perkin Elmer). Using CalcuSyn software (Biosoft, Ferguson, MO), results were analyzed for synergistic, additive, or antagonistic effects. Synergism is indicated by a Combination Index (CI) of < 0.9, additivity by CI values of 0.9-1.1, and antagonism by CI > 1.1 [31]. To determine the IC_50 _for NVP-BEZ235, we used XLfit software (IDBS, Surrey, UK).

### Immunoblots

After treatment with NVP-BEZ235 at 100 ηM for 1, 6 and 24 hours, cells were lysed using standard methods. Primary rabbit anti-human antibodies were used: phosporylated AKT Ser^-473^, phosphorylated p70S6K Thr^389 ^and phosphorylated pS6 Ser^235/236 ^at 1:1000 (Cell Signaling Technologies). To assess apoptosis, cells were treated with 100 ηM, 500ηM and 1000 ηM NVP-BEZ235 for 72 hours. Levels of cleaved PARP (rabbit polyclonal antibody, Cell Signaling) and cleaved caspase-2 (mouse monoclonal antibody, BD Biosciences) were measured at 1:1000 for both. Mouse or rabbit anti-β-actin antibodies (Sigma Aldrich) were used to visualize protein gel loading.

## Results

### AQUA analyses

**Figure 1:**
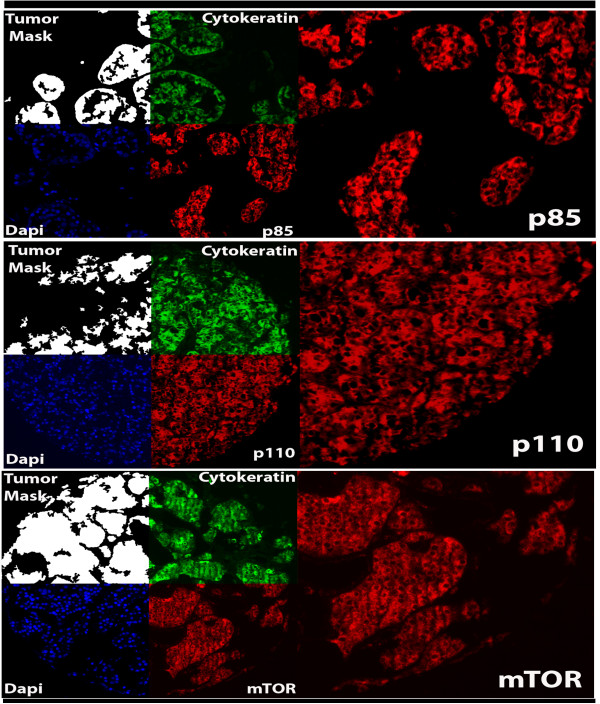
**Automated, Quantitative Analysis (AQUA) of expression of p85, p110α and mTOR in renal cell carcinoma**. AQUA uses cytokeratin to create a tumor mask (two upper left quadrants at × 10). Cytokeratin staining was cytoplasmic and the mask is made by filling in holes (lower left quadrants on left). 4', 6-diamidino-2-phenylindole (DAPI) defines the nuclear compartment within the tumor mask, which is then subtracted from the tumor mask to create a cytoplasmic compartment within the tumor mask. Target expression (**A **- p85, **B **- p110α and **C **- mTOR) expression is measured within the cytoplasmic compartments, within the tumor mask (lower right quadrants), and each clinical case is assigned a score based on pixel intensity per unit area within the tumor mask. Squares on right show × 40 magnification. in the tissue microarray. The correlation with p110α was particularly strong.

To assess intra-tumor heterogeneity, two separate slides, each containing a core from a different area of the tumor for each patient, were used for each marker (p85, p110α and mTOR). None of the markers had nuclear staining, and only membranous/cytoplasmic compartments were analyzed. By log-regression analysis, scores for matching histospots were highly correlated (R = 0.7 for p85, R = 0.8 for p110α and R = 0.7 for mTOR). Scores from the automated analysis are continuous from 0 to 255. The range of AQUA scores was 3.6-91.4 (median-32.3) for p85, 1.8-46.5 (median-7.9) for p110α and 4.1-75.5 (median-25.38) for mTOR. Examples of strong AQUA staining for p85, p110α and mTOR are shown in Figure 1A-C.

Scores from the two slides were combined for a single dataset. Spots were deemed uninterpretable if they had insufficient tumor, loss of tissue or abundant necrosis. A composite score was formed by averaging the scores. Patients with only one core were excluded from the analysis. The combined dataset had 264 cases for p85, 237 for p110α and 267 for mTOR.

**Figure 2:**
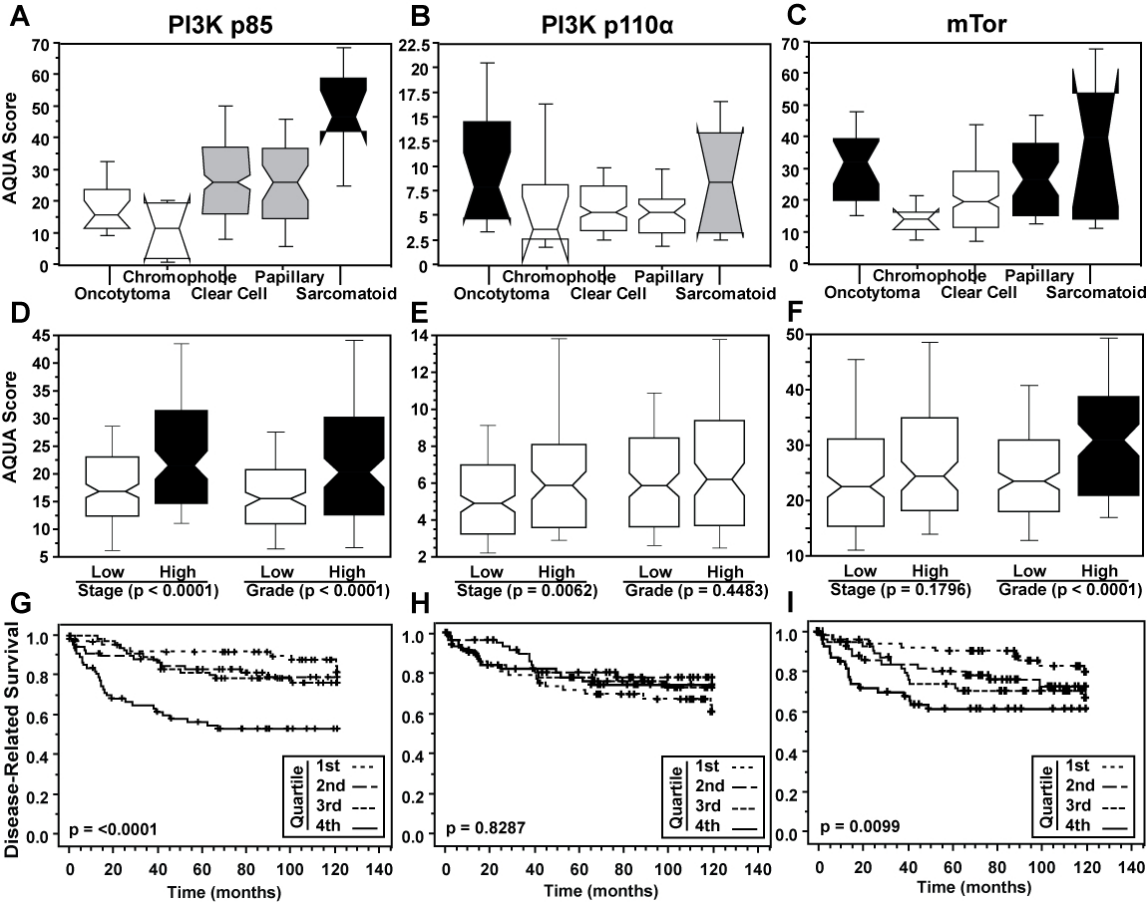
**Associations between marker expression and clinical/pathological variables**. **(A**) Associations between target expression (p85, p110α and mTOR) and histologic subtype. Expression of all three targets was higher in sarcomatoid tumors. **(B) **Associations between target expression and tumor stage and Fuhrman grade. p85 and mTOR were associated with high grade and p85 with high stage. p110α levels were not associated with stage or grade. **(C) **Kaplan-Meier survival curves of quartiles of AQUA scores for the three targets. High levels of both p85 and mTOR were associated with decreased survival.

We found a moderate correlation between expression of the two PI3K subunits (ρ = 0.129, *P *= 0.046) and stronger correlations between mTOR and the two PI3K subunits; ρ = 0.251 for p85 and ρ = 0.385 for p110α (*P *< 0.0001 for both). Expression of both PI3K sub-units and mTOR was significantly higher in sarcomatoid tumors (*P *= 0.002, *P *= 0.04 and *P *= 0.02, respectively), and expression of p110α and mTOR was also significantly higher in oncocytomas. Expression of mTOR was also somewhat higher in papillary carcinomas (*P *= 0.02) (Figure 2A). We found significant differences in p85 expression between early and late stage disease, and expression of mTOR was higher in high grade tumors), (Figure 2B), (*P <*0.0001 for all). p85 expression was higher in cases with high Fuhrman grade (*P *< 0.0001) (Figure 2B). No association was found between expression of p110α and stage or grade **(**Figure 2B).

AQUA provides continuous output scores rather than divisions into "high" and "low" categories. We therefore arbitrarily divided the continuous AQUA scores for the three markers into quartiles. For p85 and mTOR, survival of patients with AQUA scores in the top quartile was significantly lower (Figure 2C). Using Cox univariate analysis of continuous AQUA scores, high p85 PI3K expression was strongly associated with decreased survival (*P *< 0.0001). No association was found between continuous p110α scores and survival (*P *= 0.8287), while continuous mTOR AQUA scores were associated with decreased survival (*P *= 0.0099).

Using the Cox Proportional Hazards Model, we performed multivariable analyses. Expression of p85 retained its independent prognostic value, as did stage and Fuhrman grade (Table 1).

**Table 1:** PI3K, p85 and p110 and mTOR Multivariate Analysis*

Marker	Relative	95% Confidence			*P*-value	
	Risk	Interval				
**PI3K - p85**	**1.026**	**[1.004**	**-**	**1.048]**		**0.02**
PI3K - p110α	0.946	[0.872	-	1.025]		0.1762
mTOR	1.017	[0.992	-	1.042]		0.178
**Stage**	**4.087**	**[2.120**	**-**	**5.469]**	**<**	**0.0001**
**Nuclear Grade**	**2.898**	**[1.536**	**-**	**5.469]**		**0.001**

*Statistically significant markers are bolded

### Synergism between PI3K and mTOR inhibition

**Figure 3:**
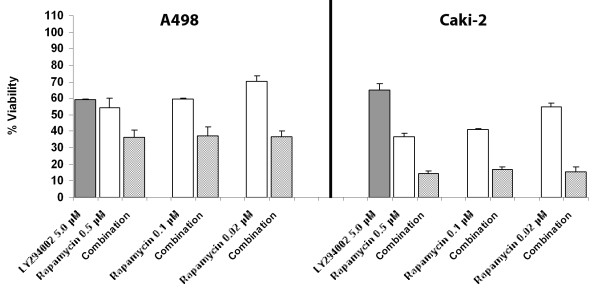
**Synergism between PI3K and mTOR inhibition**. Cell viability assays in A498 **(A) **and Caki-2 cells **(B): **Cells were treated with LY294002 alone, rapamycin alone (at three concentrations) or the combination of LY294002 and rapamycin. The combinations were highly synergistic, with similar viability seen for all three concentrations of rapamycin used. There was no significant difference between the different combination therapies (*p *> 0.5 for all).

Using 5, 25 and 50 μM of LY294002, we studied synergism with a range of concentrations of rapamycin (20, 100 and 500ηM). Synergism was seen in all six cell lines at 5 μM LY294002 with all three concentrations of rapamycin (Table 2). We note that the degree of viability inhibition with all concentrations of rapamycin was almost identical, as shown in Figure 3, using A498 and Caki-2 cells as examples (p > 0.5 for comparison between combinations of rapamycin and LY294002). Viability of cells treated with LY294002, rapamycin or the combination is calculated as a percent of the viability of the untreated (control) cells.

**Table 2:** Combination indexes assessing synergism/additivity/antagonism in rapamycin and LY294002

LY294002	Rapamycin	A498	ACHN	CAKI-1	CAKI-2	769-P	786-O
(nM)	(nM)	(CI)	(CI)	(CI)	(CI)	(CI)	(CI)
5000	500	0.476	0.371	0.593	0.201	0.430	0.242
5000	100	0.461	0.193	0.492	0.212	0.444	0.262
5000	20	0.441	0.173	0.472	0.184	0.385	0.281
25000	500	1.388	0.678	1.409	1.270	1.356	0.830
25000	100	1.420	0.586	1.193	1.074	1.430	0.847
25000	20	1.393	0.484	1.227	1.020	1.530	0.910

Synergism is indicated by a CI of < 0.9, additivity by CI values between 0.9 and 1.1, and antagonism by CI of > 1.1.

### Activity of the dual PI3K-mTOR inhibitor NVP-BEZ235 in RCC cell lines

Given the synergism seen between the LY294002 and rapamycin in RCC cell lines, we studied the *in vitro *activity of NVP-BEZ235, which has been given to solid tumor patients in phase I clinical trials. In all 6 RCC cell lines the IC_50_s of this compound were in the ηM range (Table 3).

**Table 3:** IC_50 _values of RCC cell lines treated with NVP-BEZ235

Cell Line	IC_50 _NVP-BEZ235 (ηM)
A498	25
ACHN	30
Caki-1	26
Caki-2	164
769-P	67
786-0	87

In all 6 RCC cell lines the IC_50_s of NVP-BEZ235 were in the ηM range.

### NVP-BEZ235 target inhibition and induction of apoptosis

**Figure 4:**
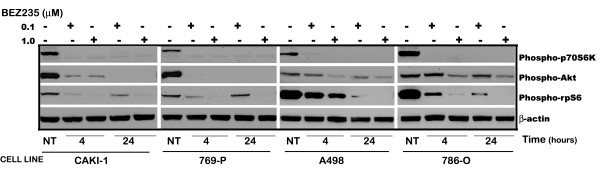
**Effects of NVP-BEZ235 on renal cell carcinoma cells *in vitro***. Targets of NVP-BEZ235, p-P70S6K, p-Akt and p-S6 were decreased in Caki-1, 769-P, A498 and 786-0 cells with exposure to the drug. Cells were exposed to 0.1 and 1.0 μM NVP-BEZ235, or DMSO (vehicle) for 4 and 24 hours. β-actin is shown as a loading control.

Targets of NVP-BEZ235, p-P70S6K, p-Akt and p-S6 were decreased in Caki-1, 769-P, A498 and 786-0 cells with exposure to the drug. Cells were exposed to 0.1 and 1.0 μM NVP-BEZ235, or DMSO for 4 and 24 hours. β-actin is shown as a loading control. p-P70S6K levels are undetectable at all concentrations and time points studied, whereas levels of p-Akt and p-S6 decrease after 4 hours of drug exposure in a dose-dependent fashion (Figure 4). Exposure of RCC cells to ascending concentrations of NVP-BEZ235 at 72 hours resulted in PARP cleavage and cleavage of caspase-2 (Additional file 1, Figure 1). Caspase-2 was selected as it has been shown in other publications to be activated in response to treatment with NVP-BEZ235 [32,33].

## Discussion

We studied expression patterns of PI3K pathway members critical for cell survival and proliferation in a large cohort of RCC specimens. We used a novel method of quantitative immunofluorescence, AQUA. This method is void of the pathologist-based bias associated with DAB staining. The p85 subunit was associated with high grade, high stage and decreased survival, and remained an independent prognostic marker on multi-variable analysis. p110α was not associated with high stage, grade or survival. mTOR was associated with survival on uni-variable analysis; however on multi-variable analysis it lost its independence as a prognostic marker. The association between PI3K and mTOR and disease progression suggests that they might be valuable drug targets. The p85 subunit has both a regulatory and a stimulatory role in activity of the PI3K pathway. The p110α subunit is thought to be stimulatory only. The functional roles of the subunits, in conjunction with our findings of stronger co-expression of the p110α subunit and mTOR, suggest that pharmacological co-targeting of p110α and mTOR might be a useful strategy for treating RCC.

Activation of the PI3K-Akt pathway and its role in RCC progression was previously evaluated in a small study of 48 patients with RCC by immunohistochemistry using an antibody to p-Akt, showing that p-Akt was associated with high tumor grade and metastatic disease. In addition, high p-Akt immunostaining was significantly associated with decreased cancer-specific survival [34]. Activation of the PI3K-Akt signaling pathway was also examined in RCC cell lines treated with PI3K inhibitors, wortmannin and LY294002 in previous studies [21]. This study demonstrated that the PI3K-Akt signaling pathway is constitutively activated in RCC cells, regardless of VHL status, and that activation of this pathway is tumor specific relative to corresponding normal renal tissue [21]. The same group conducted *in vivo *studies of nude mice bearing human RCC xenografts treated with LY294002. LY294002 inhibited tumor growth, and p-Akt was reduced in these tumors [21].

The recognition that the PI3K pathway has gained as a putative target in cancer therapy is reflected by the recent increase in literature regarding novel PI3K inhibitors [4,15,35-37]. Preliminary data from a phase I study of the oral PI3K/mTOR inhibitor, NVP-BEZ235 was conducted in patients with histologically confirmed, advanced, unresectable solid tumors[38]. The findings in the breast and colorectal patients which were reported showed that NVP-BEZ235 was well tolerated with a favorable safety profile.

There is also emerging evidence that mTOR activation may play a role in promoting cell survival through the activation of antiapoptotic proteins that contribute to tumor progression. Given that the mTOR pathway is deregulated in a number of cancers, it was anticipated that mTOR inhibitors would have broad therapeutic application across many tumor types. Two mTOR inhibitors have been approved for use in metastatic RCC. Both have clinical activity in this disease, however primary and acquired resistance limit their use, and our studies suggest that the addition of a PI3K inhibitor might result in improved outcome. While both wortmannin and LY294002 have provided tools to study PI3K inhibition in pre-clinical models, the clinical use of these compounds is limited due to their chemical properties, lack of specificity and poor tolerability [39-41]. Given the diversity of activity of PI3K family members, isoform-selective inhibitors could potentially be better tolerated [42]. Compounds that inhibit the p110*α *and p85 subunits with a high degree of selectivity are in development. Examples include the semi-synthetic viridin and wortmannin derivative PX-866 (Oncothyreon/ProIX Pharmaceuticals) which has entered Phase I trials, the LY294002 RGDS-conjugated pro-drug SF-1126 (Semafore Pharmaceuticals) which has entered Phase I/II trials [43]. GDC-0941 (Genentech/Piramed/Roche) is a Pan-class I PI3K inhibitor in Phase I trials. The Exelexis compounds XL-147 and XL-765 are also in Phase I trials.

In our models, activity of LY294002 alone in RCC cell lines was limited, with IC_50_s in the micromolar range. While this compound is also a weak inhibitor of mTOR, there are a number of potential mechanisms of resistance to PI3K inhibitors when administered alone. For example, Akt can be activated by PI3K-independent mechanisms such as mTORC2 activation [44]. Members of the MAPK pathway have been shown to activate Akt as well: ERK and RSK inhibit TSC2, which can result in mTOR activation despite effective PI3K inhibition, as reviewed [45]. Others have shown that inhibition of PI3K results in down-regulation of S6K, a negative regulator of PI3K through phosphorylation and inhibition of insulin receptor substrate 1, causing a negative feedback loop, as reviewed by Chalhoub and Baker [46]. One potential method to overcome this resistance to pure PI3K inhibition is co-inhibition of the down-stream mediator, mTOR.

We found that the combination of LY294002 and rapamycin was highly synergistic in all six RCC cell lines studied. We used concentrations of rapamycin that ranged from 20ηM to 500ηM. Similar inhibition of viability was seen with all rapamycin concentrations used. This is most important when designing novel therapies and novel drug combinations, particularly as toxicity associated with higher doses of mTOR inhibitors can be quite remarkable [12]. Grade 3 adverse events occur in a subset of patients treated with temsirolimus monotherapy and include hematologic toxicities, hyperlipidemia, hyperglycemia, asthenia and dyspnea. Similar toxicities were seen in patients treated with everolimus [47]. Moreover, combinations of mTOR inhibitors and other targeted therapies have sometimes been surprisingly toxic [48].

Due to the poor pharmacologic properties of LY294002, we further investigated the co-targeting of PI3K and mTOR using a clinical-grade dual inhibitor, NVP-BEZ235. Previously, significant toxicity in preclinical models has been an issue in combined PI3K and mTOR inhibitor studies. NVP-BEZ235 has an advantageous pharmacologic profile and in vivo administration results in high and sustained exposure in tumor tissue [49]. It inhibits both mTORC1 and mTORC2, resulting in enhanced inhibition of p-Akt compared to either LY294002 or rapamycin, or the combination of LY294002 and rapamycin, as shown in other malignancies [50]. We found that this compound was highly active *in vitro*, inhibiting RCC cell growth with IC_50_s in the low ηM range. Our studies further support results published by Cho et. al demonstrating growth arrest in RCC cell lines in vitro and in vivo using NVP-BEZ235 [51].

## Conclusion

Expression of PI3K and mTOR is upregulated in aggressive RCC tumor cells, suggesting that these are valuable drug targets. Co-expression of the p110α subunit and mTOR further indicate that co-targeting these molecules in RCC might be a useful therapeutic approach. We found that concurrent use of PI3K and mTOR targeting drugs in RCC cell lines was synergistic in all cell lines studied. The dual PI3K/mTOR inhibitor NVP-BEZ235 that is currently in clinical development is highly active in RCC models, and further evaluation of this compound in RCC is warranted.

## Funding

AAE is supported by a Young Investigator Award from the American Society of Clinical Oncology. RLC is supported by NIH Grant R21 CA116265. HMK is supported by NIH grants RO-1 R0-1 CA158167 (to H. Kluger) R0-1 CA129034 (to F. Waldman) and by American Cancer Society Award M130572 (to H. Kluger).

## List of abbreviations

The following abbreviations were used: AQUA: Automated; Quantitative Analysis; DAPI: 4; 6-diamidine-2-phenylindole; HIF: hypoxia-induced factor; mTOR: Mammalian target of Rapamycin; PI3K: phospahtidylinositol-3 kinase; RCC: renal cell carcinoma; RTK: receptor tyrosine kinase; and TMA: tissue microarray.

## Competing interests

RLC is a co-founder, stockholder and consultant for a company called HistoRx that has licensed the technology for automated tissue analysis used in this study. WH and MM are employees and stock-holders of Novartis Pharmaceuticals. AE, SAA, PJC, SS and HMK have no competing interests.

## Authors' contributions

AAE, SAA and PJC performed experiments. AAE and HMK designed experiments. AAE, HMK and SAA wrote the manuscript. RLC performed the statistical analysis. HMK supervised the project. All authors read and approved the final manuscripts.

## Supplementary Material

Additional file 1 **Induction of apoptosis by NVP-BEZ-235**. Western blots demonstrating caspase-2 induction and PARP cleavage in RCC cells exposed to NVP-BEZ-235 Click here for file
