# Supplementary material for: Characterization and targeting of phosphatidylinositol-3 kinase (PI3K) and mammalian target of rapamycin (mTOR) in renal cell cancer
Source: J Transl Med. 2011 Aug 11;9:133. doi: 10.1186/1479-5876-9-133 (PMC3173341; doi:10.1186/1479-5876-9-133)
Supplement: Additional file 1 — Induction of apoptosis by NVP-BEZ-235. Western blots demonstrating caspase-2 induction and PARP cleavage in RCC cells exposed to NVP-BEZ-235 [file 1479-5876-9-133-S1.DOC]

**Supplemental Figure 1:** Western blots showing increased levels of cleaved PARP (indicating increased apoptosis) and decreased levels of pro-caspase-2 (the inactive form of caspase-2) in 769P cells with no treatment and with exposure to 100 ηM, 500 ηM and 1000 ηM of NVP-BEZ235 for 72 hours, indicating dose-dependent induction of apoptosis.

**
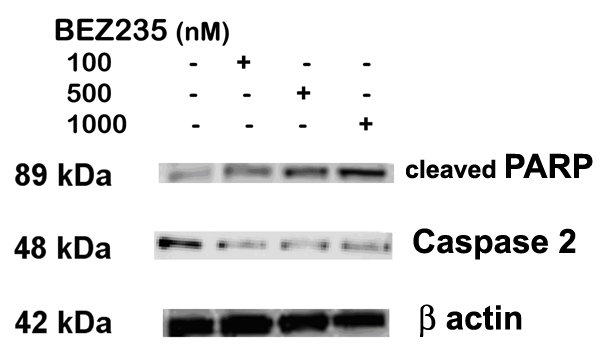
**
